# Supplementary material for: Superior Efficacy of Apathogenic Genotype I (V4) over Lentogenic Genotype II (LaSota) Live Vaccines against Newcastle Disease Virus Genotype VII.1.1 in Pathogen-Associated Molecular Pattern-H9N2 Vaccinated Broiler Chickens
Source: Vaccines (Basel). 2023 Oct 25;11(11):1638. doi: 10.3390/vaccines11111638 (PMC10674370; doi:10.3390/vaccines11111638)
Supplement: Supplementary file 1 [file vaccines-11-01638-s001.zip › vaccines-2587246-supplementary tables and figures.pdf]

Supplementary Table S1: Mortality rates and clinical disease scores in all groups for 10 days post-infection

Groups (15 birds each)	Days Post Challenge										Total Mortality	Clinical disease score
	1	2	3	4	5	6	7	8	9	10		
1	0	0	0	0	0	0	0	0	0	0	0	0.1±0.01[d]
2	0	0	1	1	1	2	1	0	0	0	6 (40%)	0.6±0.06[c]
3	0	2	2	5	4	1	1	NA	NA	NA	15 (100%)	1.5±0.09[a]
4	0	0	0	0	0	0	0	0	0	0	0 (0%)	0.0±0.00[d]
5	0	0	0	0	0	0	0	0	0	0	0 (0%)	0.1±0.01[d]
6	0	0	1	2	4	0	0	0	0	0	7 (46.66%)	0.8±1.0[b]
7	0	3	5	5	2	NA	NA	NA	NA	NA	15 (100%)	1.6±0.06[a]
8	0	0	0	0	0	0	0	0	0	0	0 (0%)	0.0±0.00[d]

NA: not applied as all birds died.

Supplementary Table S2: Clinical disease scores in all groups

Chicken group	Clinical signs	Days after inoculation<br>Number of chickens with specific signs										Total score
		1	2	3	4	5	6	7	8	9	10	
G1	Normal	15	15	15	12	10	15	15	15	15	15	142 × 0 = 0
	Sick	0	0	0	3	5	0	0	0	0	0	8 × 1 = 8
	Dead	0	0	0	0	0	0	0	0	0	0	0 × 2 = 0
	Total score = 8/150 = 0.1											
G2	Normal	0	0	12	10	7	5	5	5	6	8	58 × 0 = 0
	Sick	0	0	2	3	5	5	4	4	3	1	27 × 1 = 27
	Dead	0	0	1	2	3	5	6	6	6	6	35 × 2 = 70
	Total score = 97/150 = 0.6											
G3	Normal	15	6	2	0	0	0	0	0	0	0	23 × 0 = 0
	Sick	0	7	9	6	2	1	0	0	0	0	25 × 1 = 25
	Dead	0	2	4	9	13	14	15	15	15	15	102 × 2 = 204
	Total score = 229/150 = 1.5											
G4	Normal	15	15	15	15	15	15	15	15	15	15	150 × 0 = 0
	Sick	0	0	0	0	0	0	0	0	0	0	0 × 1 = 0
	Dead	0	0	0	0	0	0	0	0	0	0	0 × 2 = 0
	Total score = 0/150 = 0											
G5	Normal	15	15	13	10	12	14	15	15	15	15	139 × 0 = 0
	Sick	0	0	2	5	3	1	0	0	0	0	11 × 1 = 11
	Dead	0	0	0	0	0	0	0	0	0	0	0 × 2 = 0
	Total score = 11/150 = 0.1											
G6	Normal	0	0	11	8	3	2	3	3	4	6	40 × 0 = 0
	Sick	0	0	3	4	5	6	5	5	4	2	34 × 1 = 34
	Dead	0	0	1	3	7	7	7	7	7	7	46 × 2 = 92
	Total score = 126/150 = 0.8											
G7	Normal	15	3	0	0	0	0	0	0	0	0	18 × 0 = 0
	Sick	0	9	7	2	0	0	0	0	0	0	18 × 1 = 18
	Dead	0	3	8	13	15	15	15	15	15	15	114 × 2 = 228
	Total score = 246/150 = 1.6											
G8	Normal	15	15	15	15	15	15	15	15	15	15	150 × 0 = 0
	Sick	0	0	0	0	0	0	0	0	0	0	0 × 1 = 0
	Dead	0	0	0	0	0	0	0	0	0	0	0 × 2 = 0
	Total score = 0/150 = 0											

Clinical signs included general signs of depression and decreased feed intake; respiratory signs, such as sneezing, rales, nasal and ocular discharge, conjunctivitis, coughing, and head swelling; enteric signs such as greenish diarrhea; nervous signs, such as head shaking, torticollis, and lateral recumbency; as well as post mortem lesions of tracheitis, pneumonia, proventricular hemorrhages, enteritis, petechial hemorrhages on ileocecal tonsils, splenitis, hepatic congestion with distended gall bladder, and nephritis.

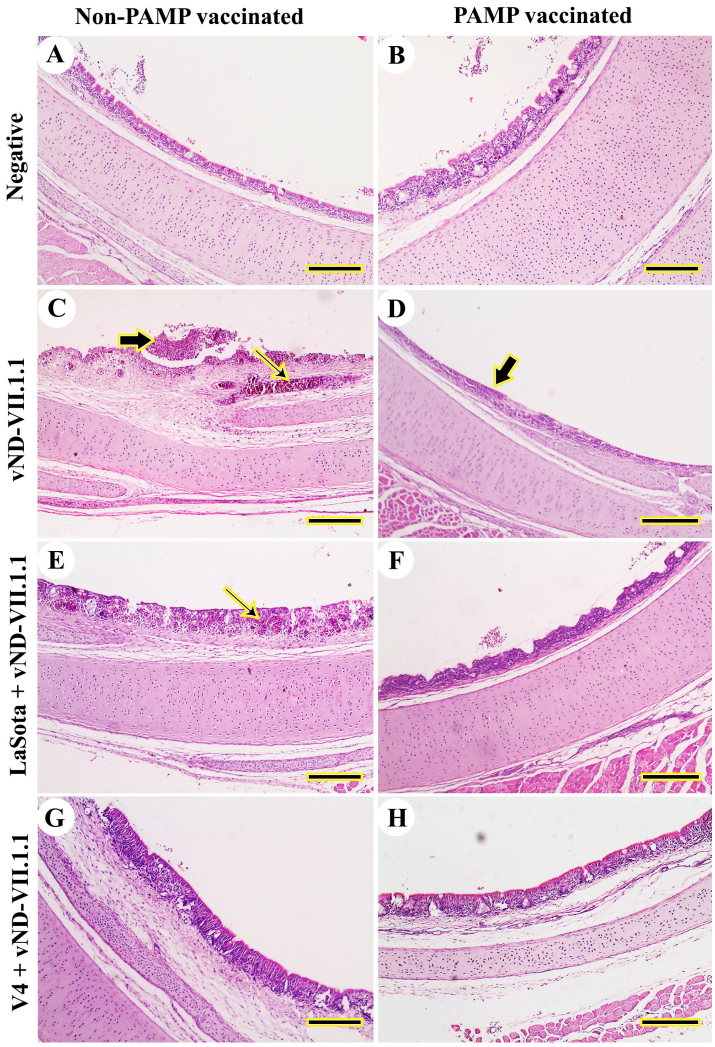

Supplementary figure S1. Histopathological examination of chicken tracheas. (**A**) Negative control (G8). (**B**) H9N2-PAMP vaccination only (G4). (**C**) vND-VII.1.1 challenge only (G7). (**D**) H9N2-PAMP vaccination and vND-VII.1.1 challenge (G3). (**E**) LaSota vaccination and vND-VII.1.1 challenge (G6). (**F**) H9N2-PAMP, LaSota vaccination, and vND-VII.1.1 challenge (G2). (**G**) V4 vaccination and vND-VII.1.1 challenge (G5). (**H**) H9N2-PAMP, V4 vaccination, and vND-VII.1.1 challenge (G1). Normal cilia (thin arrows), hemorrhage (arrowheads), and mucosal hyperplasia and metaplasia (thick arrows). Scale bar = 200 μm.

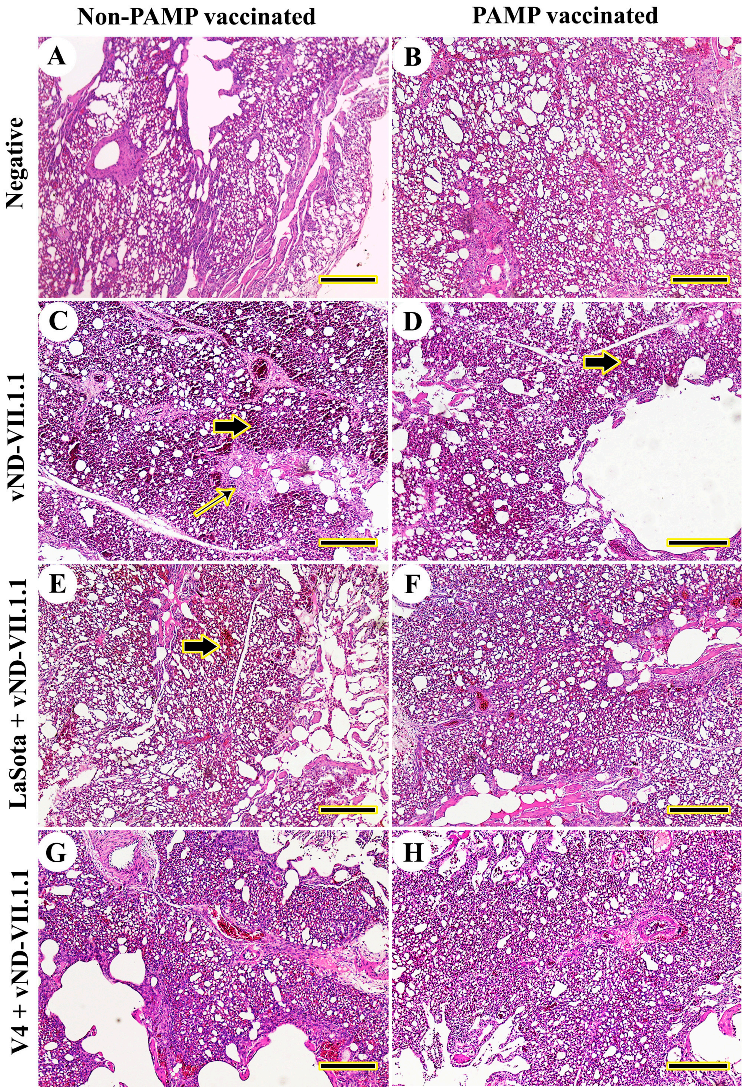

Supplementary figure S2. Histopathological examination of chicken lungs. (**A**) G8. (**B**) G4. (**C**) G7. (**D**) G3. (**E**) G6. (**F**) G2. (**G**) G5. (**H**) G1. hemorrhage (thick arrows), and thick interstitial connective tissue filled with inflammatory cells (thin arrows). Scale bar = 200 μm.

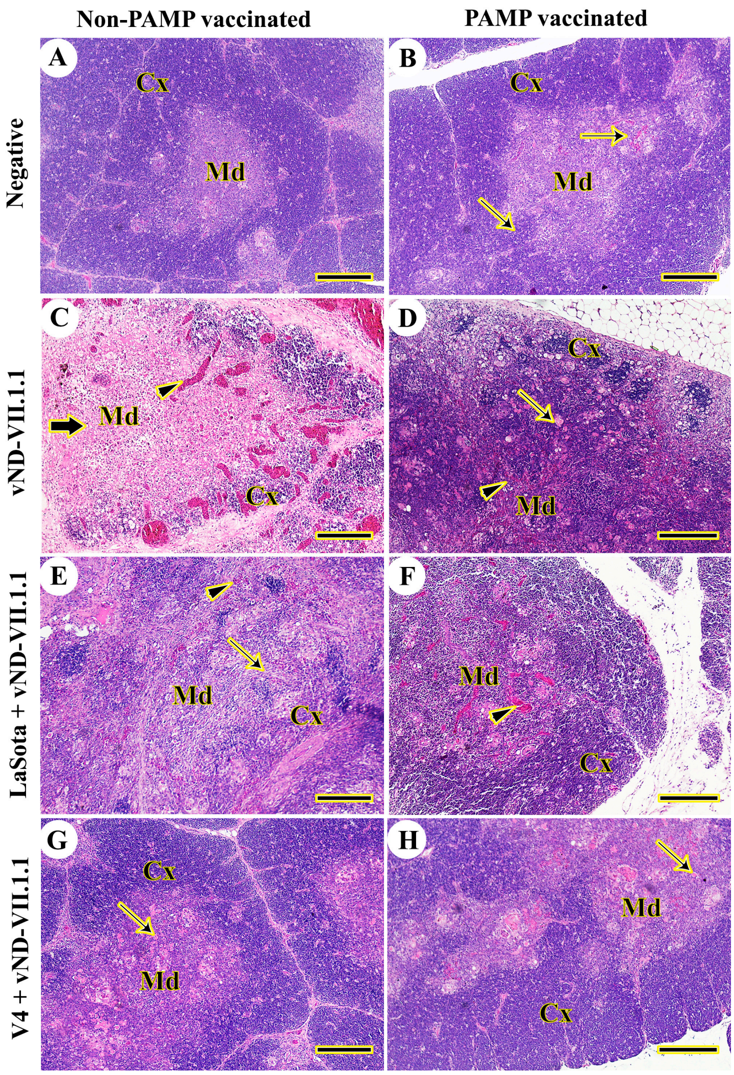

Supplementary figure S3. Histopathological examination of chicken's thymus glands. (**A**) G8. (**B**) G4. (**C**) G7. (**D**) G3. (**E**) G6. (**F**) G2. (**G**) G5. (**H**) G1. Cortex (Cx), medulla (Md), Hassell's corpuscles (thin arrows), severe congestion (arrowheads), and necrosis (thick arrows). Scale bar = 200 μm.

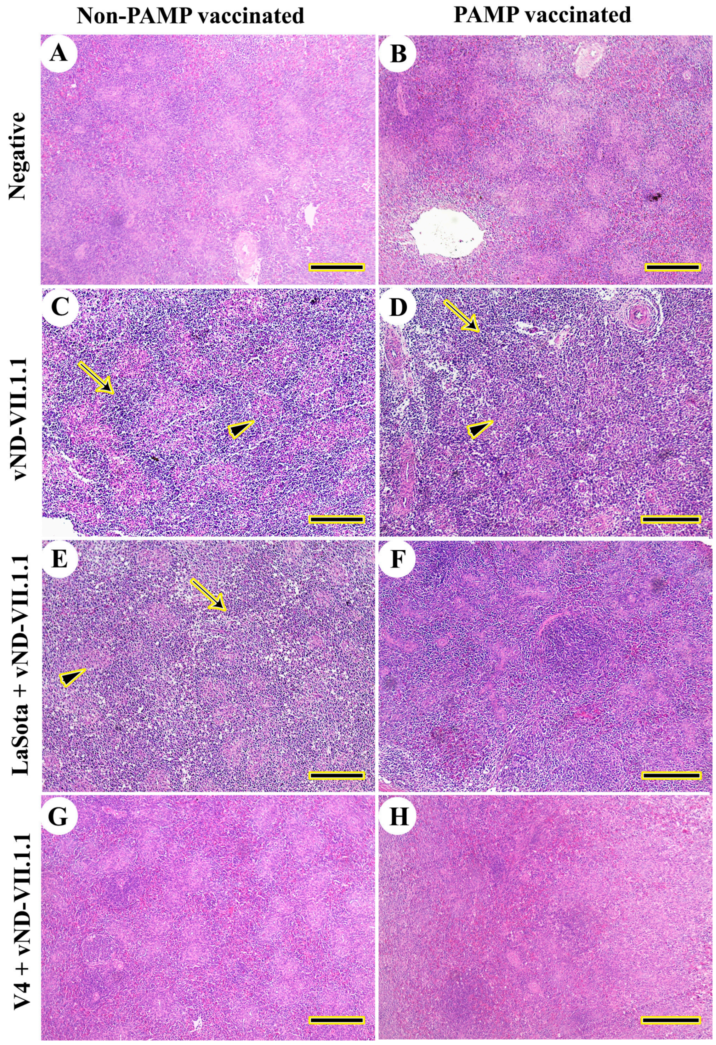

Supplementary figure S4. Histopathological examination of chicken spleens. (**A**) G8. (**B**) G4. (**C**) G7. (**D**) G3. (**E**) G6. (**F**) G2. (**G**) G5. (**H**) G1. Necrosis (thin arrows), and multifocal lymphoid depletion (arrowheads). Scale bar = 200 µm.

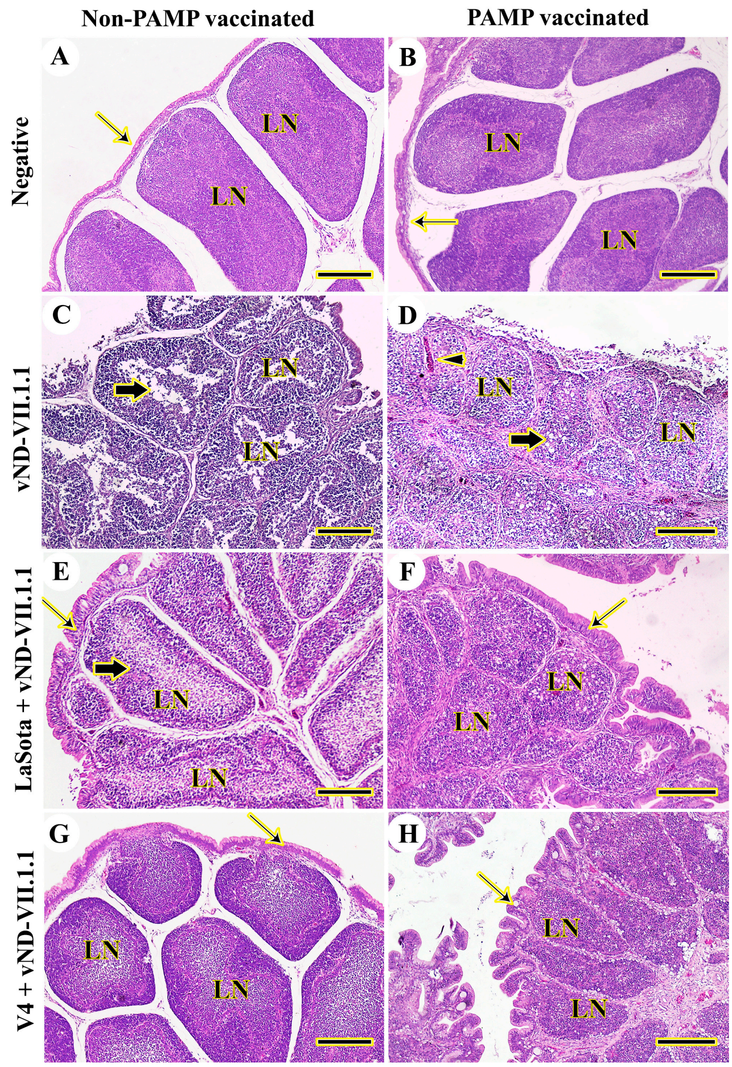

Supplementary figure S5. Histopathological examination of chicken bursa of Fabricius. (**A**) G8. (**B**) G4. (**C**) G7. (**D**) G3. (**E**) G6. (**F**) G2. (**G**) G5. (**H**) G1. Lymphoid nodule (LN), normal epithelium (thin arrows), necrosis and lymphoid depletion (thick arrows), and congested blood vessels (arrowheads). Scale bar = 200 µm.
